# NO_2_ Detection Using Microcantilever Based Potentiometry

**DOI:** 10.3390/s8117144

**Published:** 2008-11-12

**Authors:** Muhammad Qazi, Goutam Koley

**Affiliations:** 1 Department of Electrical Engineering, University of South Carolina, Columbia, SC29208, USA; 2 USC Nanocenter, University of South Carolina, Columbia, SC29208, USA; E-Mail: koley@engr.sc.edu (G. K.)

**Keywords:** Microcantilever, potentiometry, surface work function, nanoscale graphite, NO_2_ sensor

## Abstract

A highly sensitive and novel sensor platform for gases and volatile chemicals using microcantilever based potentiometry is reported. A resonant cantilever is used to detect the changes in surface work functions of functionalized substrates caused by adsorption of target gas molecules. Surface work function (SWF) changes were measured for different functionalization layers made of transition metal oxide thin films with the flow of NO_2_. The rate of change in SWF for In_2_O_3_ and SnO_2_ were found to be ∼80 and ∼100 μV/sec, respectively, for 70 ppm NO_2_. A sensitivity of 64 μV/sec for SWF change was also found for 70 ppm NO_2_ concentration for isolated clusters of ZnO nanowires, indicating that this technique is applicable even for nano-clusters of sensing materials where amperometric detection is impossible due to material discontinuity. NO_2_ detection as low as 400 ppb was possible using highly insulating In_2_O_3_ and SnO_2_ thin films (resistivity > 1 TΩ/□. Two different forms of nano scale graphite were compared with the transition oxide based functionalization layer for sensing sub-ppm NO_2_ sensing. It was observed that nanostructured graphite (NG) shows much higher sensitivity and lower response time than transition metal oxides.

## 1. Introduction

In the recent past, a great deal of research has been directed towards fast, accurate and highly sensitive gas sensing devices that are important for various applications including providing precaution against toxic gases that create health hazards, for monitoring and reducing pollutants in the environment, early detection of nerve gases in defense and military applications, and for monitoring pollutants emitted from vehicular exhaust system. Such sensors should be capable of monitoring the concentrations of particular gases continuously with accuracy and selectivity [1]. Nitrogen oxides (mainly NO and NO_2_, together referred to as NO_x_) are among the six most common air pollutants defined by EPA (Environmental Protection Agency), along with particle pollution, ground-level ozone, carbon monoxide, sulfur oxides and lead [2]. During NO_x_ emission, NO_2_ is produced by the further oxidation of NO and the resulting concentrations of NO_2_ are rather complex. NO_2_ plays a major role in atmospheric reactions that produce ground-level ozone, a major component of smog, and it is also a precursor to nitrates, which contribute to increased respiratory problems. Continued or frequent exposure to NO_2_ concentrations higher than the EPA air quality standard (53 ppb) may cause increased incidence of acute respiratory illnesses in children [2]. NO_2_ is also one of the main toxic components emitted from vehicular exhaust and also a main component of emissions from indoor appliances. In addition, it transforms in the air to form gaseous nitric acid and toxic organic nitrates, hence contributing to the production of acid rain [3]. Therefore a selective and accurate NO_2_ sensor is of extreme importance for continuous monitoring of emission processes. Existing gas detector devices use non-selective techniques based on responses that detects the change in electronic or mechanical property of the chemoselective sensing materials due to adsorption of target molecules. One of the most common methods for detection of gases is based on the change in current flow in a semiconductor device (called an amperometric technique, since it is based on the change in current conduction due to surface adsorption upon exposure to the target gases). Examples of other available detection techniques include chem-Field Effect Transistor (FET) type devices that are based on the change in current flow [4], potentiometric sensors where changes in surface work function are measured [5,6], microcantilever based sensors where changes in static deflection [7] or frequency shifts of the cantilever are detected [8], and chemi-capacitive sensors that are based on changes in the permittivity of the dielectric medium [9]. Although widely used for detection of various gases and vapors, one of the drawbacks of the mentioned amperometric technique is that the sensing response depends on the bulk electronic properties as well as the thickness of the sensor material coating. The extent of current change, and hence the sensitivity, depends on the bulk mobility of the carriers (which in turn depends on the material properties such as the barrier height of the grain boundary [10]), as well as the thickness of the sensing film. Thus, a large variation in sensitivity and stability for different films is possible [6].

Recently, several reports have shown that microcantilever based detection is an attractive alternative to improve detection sensitivity [7, 8]. These include micro(nano)cantilevers as sensor elements for specific chemicals [7, 8, 11, 12] and biological molecules [13, 14] exploiting their high sensitivity toward changes in surface stress. A compelling feature of microcantilever sensors is that they can be operated in air, vacuum, or liquids, which facilitates the selective and sensitive detection of airborne, waterborne and pathogenic substances [15]. The microcantilever surface is usually functionalized (coated with an appropriate selective layer) to facilitate adsorption of target molecules which changes the surface stress. However, functionalization of cantilevers has several drawbacks that hinder wide commercial application of cantilever based sensors. Firstly, surface stress is extremely sensitive to uniformity and chemical integrity of the coating that can lead to sensitivity variation from cantilever to cantilever. Secondly, fabrication of series of functionalized cantilever arrays on micron scale is quite expensive and inconvenient. Thirdly, there are several molecules which adsorb on the sensing layers through exothermic or endothermic reactions creating a resultant temperature change on the cantilever [5]. This can result in unwanted heat induced expansion/contraction thereby introducing erroneous results.

As sensing materials, semiconducting transition metal oxides have been widely used for NO_2_ detection. Researchers have successfully reported NO_2_ detection using a wide range of transition metal oxides including In_2_O_3_ [16], SnO_2_ [17] and WO_3_ [18]. NO_2_ target molecules accept electrons from the transition metal oxides which creates changes in the electrical properties of the material. Carbon has also showed itself to be an ideal sensing material due to its excellent molecular adsorption property [19]. Carbon based sensors, especially those utilizing carbon nanotubes (CNT), have been the focus for gas sensing during the last decade because of their high surface-to-volume ratio, excellent mechanical stiffness and hollow structure [20-23]. However, there are still significant challenges in large scale production and deployment of CNT based sensors. Carbon based sensors have been developed and researched almost exclusively using CNTs until the discovery of graphene in late 2004 [24, 25]. Graphene based functionalization layers have been shown to have molecular level sensitivity where one electron conductance change is reported to be detected with the adsorption of single NO_2_ molecule [26]. However, the search for a highly sensitive carbon based functionalization layer that is easy to fabricate and economical to commercialize have still not concluded because of the lack of film control for graphene preparation.

**Figure 1.**
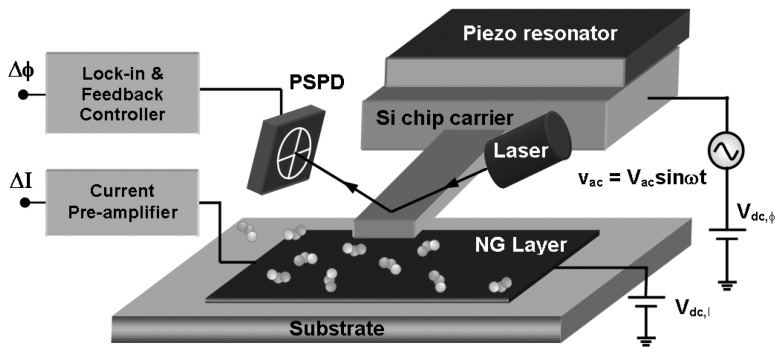
Schematic diagram of the surface work function based NO_2_ detection setup.

In this article, we demonstrate a highly sensitive microcantilever based detection technique that is generally applicable for the detection of gases and volatile chemicals, and eliminates the need for modifying the cantilever itself with a selective layer. The technique is based on adsorption induced surface work function (SWF) change of the sensing layers; however unlike other SWF based sensors [27] it uses a microcantilever which is resonated very near to a functionalized substrate with a setup similar to Kelvin probe technique (Figure 1) [5, 28]. In this work detailed analyses of the operation and sensitivity of our microcantilever based detection technique is presented. SWF changes of different transition metal oxide thin films (Section 3.1) and carbon based layers (Section 3.2) in response to trace NO_2_ are also compared. Simultaneous potentiometric and amperometric measurements on nanostructured graphite (NG) layers are discussed at the end of the article in Section 3.3.

## 2. Experimental

Microcantilever based potentiometric measurements were carried out using a commercial Atomic Force Microscope (AFM) setup (Autoprobe M5, originally made by Thermomicroscope, now a part of Veeco Metrology Group) [5]. The topographic signal was used for monitoring the cantilever oscillation. A Si microcantilever (purchased from Mikromasch, model CSC12/tipless) was excited at its resonance frequency, ω_0_ in non-contact mode using the piezo-actuator. The cantilever is excited by an external AC bias with a frequency ω which is close to ω_0_, so that beats are formed in the oscillation amplitude, and the topography feedback signal is sinusoidal (Figure 1).

The amplitude of the sinusoidal topography signal [having frequency (ω ∼ ω_0_) and representing the movement of the piezo actuator in response to the ac excitation] was recorded using the system software, which varied with change in surface work function as the target molecules get adsorbed on the functionalization layers. The amperometric measurements were performed using a current preamplifier (DL Instruments, model 1211), with metal press contacts established at the two ends of the nanostructured graphite film. The resonance frequency of the cantilevers used was ∼20 kHz with a quality factor of ∼35. A tipless cantilever is used to enhance the capacitive interaction between cantilever and surface. For our detection experiments, the desired NO_2_ concentrations were prepared by intermixing calibrated commercial NO_2_ with purified N_2_ at specific ratios using mass flow controllers (MKS Instruments). The test gas was passed at the rate of ∼200 sccm using a gas flow fixture with cross-sectional area of 1.5 cm^2^ which was positioned within a few mm of the cantilever. The cantilever-sample (electrode) distance during measurements was kept ∼10 μm during measurements. The measurement set up with an external ac bias, piezo-actuator, and the gas flow tubes are shown schematically in Figure 1.

For sensing experiments, the transition metal oxide (In_2_O_3_ and SnO_2_) thin films were deposited on a conducting Si wafer by the Successive-Ionic-Layer-Adsorption-and-Reaction (SILAR) technique followed by high temperature annealing. The SILAR process we use for depositing thin films has been described in greater detail elsewhere [29]. ZnO nanowires (the method of preparation is described in ref 30) were also investigated that were deposited on Si substrate after sonication. Clusters of nanowires were formed on the substrate after the solution dried out [Figure 3(d)]. The NG functionalization layers were prepared by mechanical exfoliation of highly pure graphite (purchased from Carbone of America Ltd.) by simple abrasion on insulating substrates. An insulating ceramic substrate was chosen to perform simultaneous amperometric and potentiometric measurements. TEM images of NG particles were obtained using high resolution Transmission Electron Microscope (Hitachi, model H-8000). The TEM samples of the mechanically exfoliated NG particles were sonicated in an acetone solution and subsequently transferred on to copper TEM grid.

## 3. Results and Discussion

The total force between the cantilever and the sensing surface consists of an electrostatic component and a capacitive component [28]. If we ignore the fixed charges on the surface the electrostatic force component can be considered zero. Therefore the total force between cantilever and the sensing surface will reduce to the capacitive component. So mathematically, if the external applied ac and dc bias is V_ac_ sin*ωt* and V_dc_ respectively, and Δφ is the surface work function difference between the cantilever and sensing surface then the total force is:

(1) $$\begin{array}{c} {\text{F}}_{\text{total}}={\text{F}}_{\text{cap}}=½\frac{\partial \text{C}}{\partial \text{Z}}{\left({\text{V}}_{\text{ac}}\sin\omega \text{t}+{\text{V}}_{\text{dc}}-\Delta \phi \right)}^{2}\\ =\left[¼\frac{\partial \text{C}}{\partial \text{Z}}{\text{V}}_{\text{ac}}^{2}+½\frac{\partial \text{C}}{\partial \text{Z}}{\left({\text{V}}_{\text{dc}}-\Delta \phi \right)}^{2}\right]+\left[\frac{\partial \text{C}}{\partial \text{Z}}\left({\text{V}}_{\text{dc}}-\Delta \phi \right){\text{V}}_{\text{ac}}\sin\omega \text{t}\right]+\left[¼\frac{\partial \text{C}}{\partial \text{Z}}{\text{V}}_{\text{ac}}^{2}\cos2\omega \text{t}\right]\\ ={\text{F}}_{0}+{\text{F}}_{\omega }+{\text{F}}_{2\omega } \end{array}$$

F_0_, F_ω_ and F_2ω_ are the dc, ω and 2ω components of the total force respectively and *∂C*/*∂z* is the capacitance gradient of the cantilever. The expression of the oscillation amplitude is given by [31],

(2) $$\text{a}=\frac{{\text{F}}_{\text{total}}}{\text{k}}\times \text{Q}$$

Here, k and Q are the spring constant and quality factor of the cantilever. As ω is very close to the resonant frequency of the cantilever, ω-component of the oscillation will be dominant due to the high quality factor of cantilever at resonant frequency. The oscillation is converted using optical transduction system and the amplitude of the ω-component is extracted by a lock-in amplifier. So the obtained electrical signal is proportional to:

$$[\overset{\partial \text{C}}{\;}/\underset{\partial \text{Z}}{\;}({\text{V}}_{\text{dc}}-\Delta \varphi ){\text{V}}_{\text{ac}}]$$

Clearly, the oscillation amplitude depends on the capacitance gradient (which in turn depends on the distance between cantilever and the substrate z), quality factor, spring constant, and the ac voltage amplitude. To analyze the sensing technique of our sensing measurements we determined the dc and ac sensitivity of the cantilever sensor.

**Figure 2.**
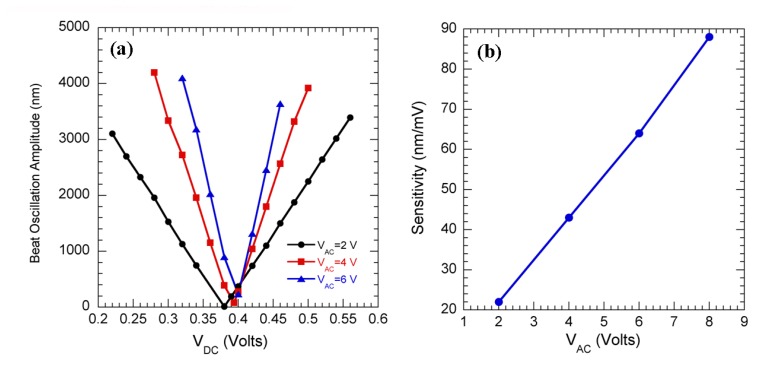
(a) Change in beat oscillation amplitude of the cantilever as a function of dc bias for 3 different ac biases, measured at a topography feedback gain of 0.6. (b) Sensitivity plotted as a function of ac bias.

The sensitivity data for uncoated Si cantilevers is shown in Figure 2(a), in which the amplitude of beat oscillations is plotted against dc bias for ac biases of 2, 4, and 6 V. Figure 2(b) shows the sensitivity plotted against the applied ac bias. The sensitivity is calculated as the rate of change of amplitude with dc bias, and expressed in nm/mV. From Figure 2(b), we see that the sensitivity increases linearly with the ac bias which directly follows from Eq. 1. The rms noise (calculated as the standard deviation of the beat amplitude as a function of time for constant ac and dc biases) corresponding to this sensitivity was found to be less than 20 nm. The maximum sensitivity obtained for our present setup was 360 nm/mV. So the minimum change in surface work function that can be resolved is calculated as 55 μV [= (20/360) mV].

We also found that the rms noise does not have any correlation with the ac bias, and varies almost randomly in the range of 8–20 nm, possibly due to the ambient noise. Increase in sensitivity with ac bias, indicates that the voltage resolution can be enhanced by increasing the ac bias. It also follows from Eq. 1 that reducing the cantilever-sample distance or spring constant or increasing Q of the cantilever will also results in increased sensitivity.

### 3.1. Transition metal oxides

**Figure 3.**
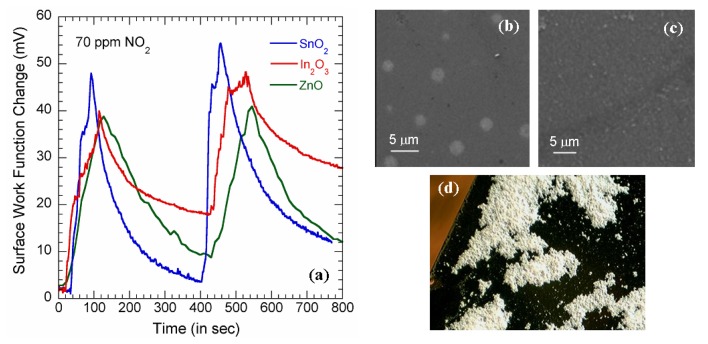
**(a)** SWF changes of In_2_O_2_, SnO_2_ and ZnO (nanowires) with 70 ppm NO_2_ flow. The gas was switched on and off for 2 cycles. **(b)** and **(c)** are the scanning electron micrographs of In_2_O_3_ and SnO_2_ thin films respectively. **(d)** 5mm×7mm optical image of clusters of ZnO nanowires spread over silicon substrate.

Measurements were performed on In_2_O_3_ and SnO_2_ thin films to investigate the surface work function responses with the adsorption of NO_2_ molecules. Figure 3(a) shows the surface work function (SWF) transients measured on In_2_O_3_ and SnO_2_ thin films when 70 ppm NO_2_ flow was turned on and off for two cycles. The oxide thin films were highly insulating (resistivity > 1 TΩ/□ which is believed to be due to the presence of high defects in the material. Such films were unsuitable for any amperometric measurements; however they showed significant response in the SWF where the initial rates of change in SWF of ∼80 and ∼100 μV/sec, respectively, were found for In_2_O_3_ and SnO_2_ [Figure 3(a)]. Figures 3(b) and 3(c) are the SEM images of the surface of the In_2_O_3_ and SnO_2_ thin films which show that they had fairly even surfaces. However, the In_2_O_3_ films show presence of tiny bubble shaped morphology with average diameter of around 3.5 μm [Figure 3(b)] that might have grown due to emission of any gaseous species during high temperature annealing. However, any error that is incorporated due these spots can be neglected since the dimension of the cantilever is relatively large (350μm × 35μm) with respect to these bubbles. To further investigate the efficacy of the potentiometric technique, we performed the NO_2_ sensing measurements on ZnO nanoclusters. ZnO has been proved as sensing material for various gases [32]; recently ZnO nanowires FET has also been demonstrated as an efficient NO_2_ sensor in sub-ppm range [33]. The ZnO nanowires we used for our experiments were deposited on Si substrate by sonication.

Figure 3(d) shows a 5 mm ×7 mm optical image of the sample produced. It can be seen in this figure that the nanowires are clustered together making small islands of material which are unsuitable as sensing materials for amerometric measurements due to the material discontinuity. However, significant SWF change was observed with flow of 70 ppm NO_2_ as the ZnO clusters showed initial rise rate of 64μV/sec [Figure 3(a)]. This shows that the potentiometric technique is equally efficient for sensing in isolated structures.

**Figure 4.**
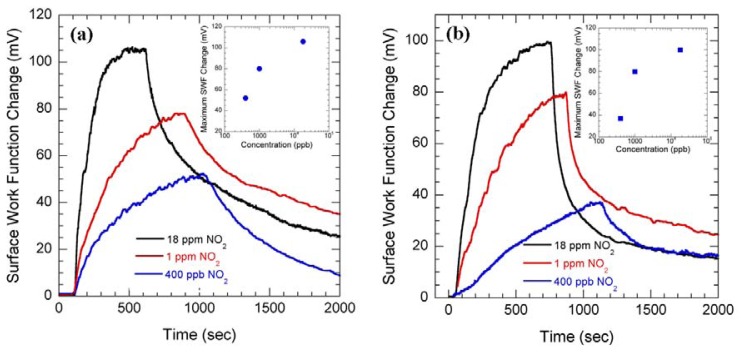
SWF changes of **(a)** SnO_2_ and **(b)** In_2_O_3_ thin films as 18, 1 and 0.4 ppm NO_2_ flow was switched on and off. Insets show maximum SWF changes plotted against the concentration of NO_2_.

The potentiometric sensing technique is also efficiently applied for NO_2_ detection in a sub-ppm regime using highly insulating SnO_2_ and In_2_O_3_ thin films. Three different concentrations of NO_2_ (18, 1 and 0.4 ppm) were switched on and off to measure the SWF change of SnO_2_ [Figure 4(a)] and In_2_O_3_ thin films [Figure 4(b)].

The observed changes in SWF for SnO_2_ thin film are ∼105, 62, and 35 mV, respectively, for 18, 1 and 0.4 ppm NO_2_, passed for 500 secs [Figure 4(a)]. The inset of Figure 4(a) shows the calibration curve for maximum SWF change plotted against the concentration of NO_2_ measured on SnO_2_ thin film. In Figure 4(b) it can be seen that after 500 secs of 18, 1 and 0.4 ppm NO_2_ flow, In_2_O_3_ thin film showed SWF changes of ∼95, 68 and 23 mV. As evident from the figures the SWF responses of In_2_O_3_ and SnO_2_ thin films with the flow of NO_2_ were very much comparable. Since the work function measurement resolution for this measurement system is ∼55 μV [5], it is also possible to further improve the NO_2_ detection sensitivity with wide area metal oxides.

### 3.2. Carbon based nano-materials

The potentiometric sensor was also applied to investigate the sensitivity of carbon based nano-scaled materials for detection of NO_2_. NG layer was deposited on insulating substrates using mechanical exfoliation technique. Insulating substrates were chosen over metallic or semiconducting surfaces to perform simultaneous amperometric and potentiometric measurements. However, the sensitivity of the potentiometric technique was found to be independent of the substrate conductivity.

**Figure 5.**
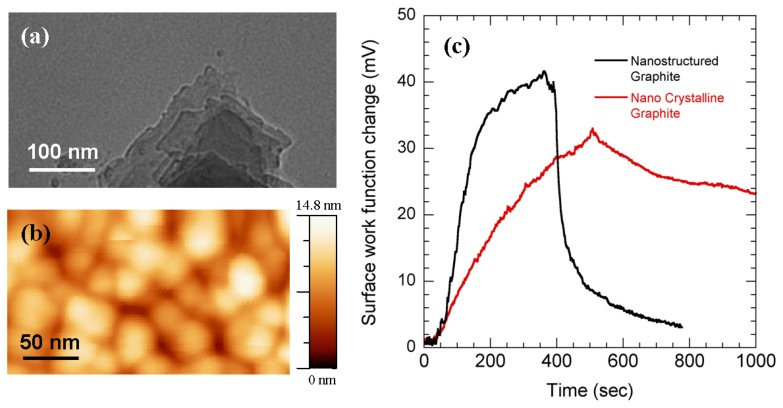
**(a)** TEM image of nanostructured graphite particles. **(b)** AFM morphological image of nanocrystalline graphite **(c)** Surface work function changes with the flow of 400 ppb NO_2_ using nanocrystalline graphite (NCG) and nanostructured graphite (NG). NG shows higher sensitivity than NCG

Figure 5(a) shows a transmission electron microscope (TEM) image of a representative NG film that we used for our sensing experiments. The image shows the presence of multi-layered graphite resembling mica-flakes, whose lateral dimensions are of the order of a few tenths of a micron. These nanostructures can significantly increase the number of the molecular adsorption sites on the surface resulting in the high sensitivity observed in our experiments. The sensitivity of the NG layers was also compared with nanocrystalline graphite (NCG) [34] layers which were prepared by Chemical Vapor Deposition (CVD) from graphitization of SiC wafers [35]. Figure 5(b) (taken from [35]) is the image of the surface morphology of NCG taken by Atomic Force Microscope (AFM) which reveals ∼20 nm grain size of the surface. However, the rms surface roughness is found to be ∼3 nm [35]. Surface work function changes of NCG and NG layers with the flow of 400 ppb NO_2_ were compared in Figure 5(c). The changes in SWF are found to be ∼25 and ∼11 mV (in 100 secs) for NG layers and NCG respectively. These responses are higher than the response we got for traditional metal oxides for sub-ppm NO_2_ (Figure 4). We believe the increased sensitivity of NG over NCG is most likely due to the increased surface roughness which increases the NO_2_ adsorption on the surface. The potentiometric sensing technique is also applied for sub-ppm NO_2_ detection using NG layers.

**Figure 6.**
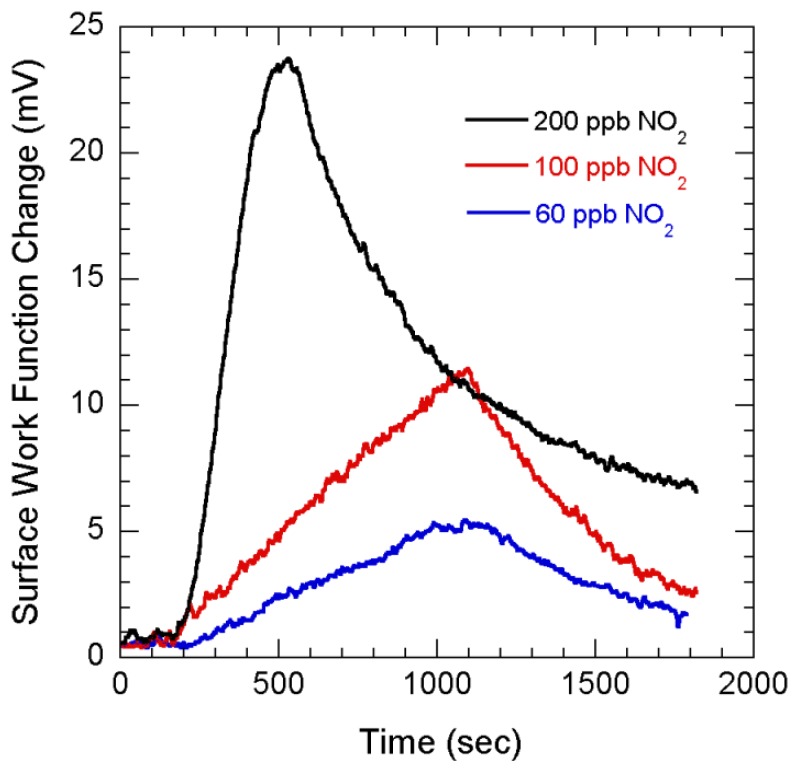
Surface work function changes of NG layers with flow of 200, 100 and 60 ppb NO_2_.

Figure 6 shows the SWF changes with flow of 200, 100 and 60 ppb NO_2_. The initial rise rates for the SWF changes in response to 3 different concentrations are found to be 80, 12 and 5 μV/sec respectively. Therefore with a noise resolution of ∼55 μV [5], we infer that 60 ppb NO_2_ can be sensed in less than 12 secs. We believe proper optimization of this sensing technique and better preparation of NG can improve the sensitivity to even lower concentration.

### 3.3. Simultaneous Amperometric and Potentiometric Measurements

**Figure 7.**
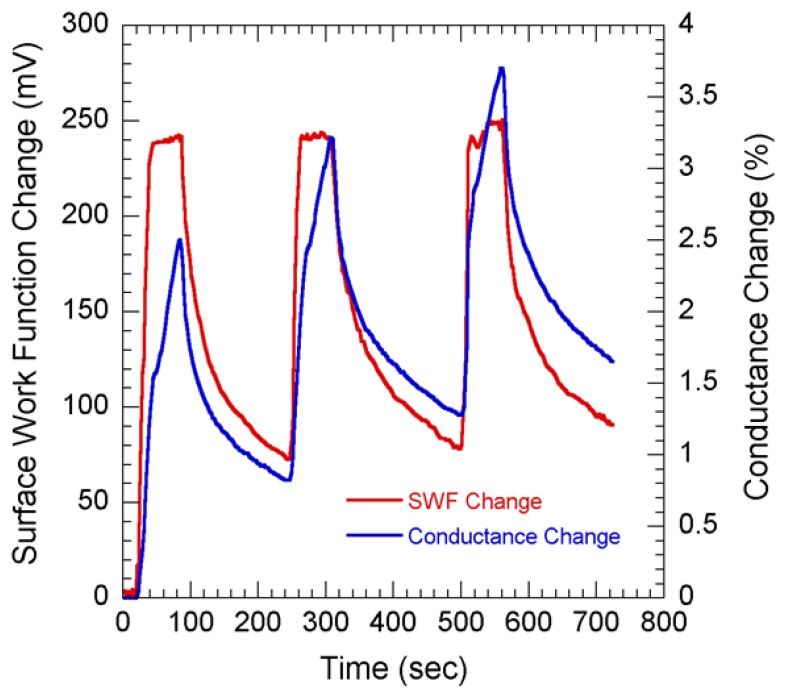
Simultaneous conductance (right axis) and surface work function (left axis) changes measured using NG layer when exposed to 100 ppm NO_2_.

The main advantage of the potentiometric technique is that it is entirely based on the surface properties of the sensing materials. On the other hand, the amperometric technique (which detects the conductance change of the material with adsorption of target molecules) relies on the adsorption process as well as the diffusive property of molecules through porous graphite. In case of molecules which do not create significant conductance change, potentiometric measurements can be a better tool since the orientation of the molecule can allow significant change in SWF. Figure 7 shows the comparison for simultaneous current and SWF measurements on NG layer with 3 cycles of 100 ppm NO_2_ flow. Carbon is a highly conductive material, so to realize the amperometric measurements, very thin layer (< 1 μm) of NG was deposited on insulating material such as ceramic.

The SWF changes quickly up to ∼240 mV within 20 secs of NO_2_ exposure; in contrast, the current increases slowly even when the SWF change had already reached saturation (Figure 7). These instances of complete surface state occupation can be distinguished as slope changes in ΔG/G (happening after ∼15 secs for each cycle). We believe that the slope change signals the end of the fast surface adsorption, which is followed by slow diffusion through the porous material and adsorption at sites deeper inside, resulting in a slow increase in current [36]. After discontinuing the NO_2_ exposure, we find that the SWF reverts to almost the original value as the molecules leave the surface, even though conductance continues to decrease slowly due to much slower desorption kinetics of the molecules from inside the porous NG material. The slow desorption causes an increase in the residual adsorbed NO_2_ molecules in successive cycles and therefore increases the minimum current reached at the end of each cycle. During the decay transient the SWF changes to ∼70 mV in ∼2.5 mins which is the same for all the 3 cycles observed. This is an indication that the surface molecules getting desorbed at the same rate during all the decay transients. However, there is less possibility of the diffused molecules to come out of the material at the same rate as the molecules getting desorbed from the surface. This is clearly observed in Figure 7 as the values of the percentage conductance changes reached 0.83, 1.3 and 1.67 respectively at the end of 3 decay transients indicating a residual increase in conduction. Figure 7 also indicates that both the SWF and conductance increases due to NO_2_ adsorption. This is possible if the graphene layer has an overall p-type nature, as has been observed in earlier studies [21]. Since NO_2_ has an electron-withdrawing nature, its adsorption enhances the p-type conductivity of the NG layer.

## 4. Conclusions

We have investigated the sensing behavior of various sensing materials for NO_2_ detection using a highly sensitive potentiometric technique. The potentiometric measurements indicate that the transition metal oxides such as In_2_O_3_ and SnO_2_ have almost similar SWF response for NO_2_ detection. The technique was found to be also sensitive for isolated structures and thin films with high resistance; these structures are unsuitable for amperometric detection. It has been found out that carbon based materials such as nanocrystalline graphite and nanostructured graphite exhibits better sensitivity than traditional transition metal oxides. The enhanced sensitivity of NG over NCG is attributed to its structural non-uniformity and increased number of adsorption site. Simultaneous SWF and conductance measurement is performed to compare the sensitivity of potentiometric technique over the amperometric one. NO_2_ detection as low as 60 ppb was possible using this technique on nanostructured graphite layers.
